# The ER-localized susceptibility factor RTP1 negatively regulates plant immunity

**DOI:** 10.1093/plphys/kiaf512

**Published:** 2025-10-14

**Authors:** Josephine H R Maidment

**Affiliations:** Assistant Features Editor, Plant Physiology, American Society of Plant Biologists; PHIM Plant Health Institute, Univ Montpellier, INRAE, CIRAD, Institut Agro, IRD, 34980 Montpellier, France; Centre de Biologie Structurale, INSERM, CNRS, Université de Montpellier, 34090 Montpellier, France

Plant pests and pathogens reduce crop yields and quality (Savary et al. 2019). Resistant crop varieties typically contain 1 or more dominant resistance genes encoding receptors that detect molecular signatures of infection. Some, however, carry mutations in susceptibility (S) genes. S genes can be involved in enabling pathogen entry/establishment, negatively regulating plant immune responses, providing the pathogen with necessary nutrition, or facilitating movement through the host plant (Van Schie and Takken 2014).

*Arabidopsis thaliana Resistance to Phytophthora parasitica 1* (*RTP1*) mediates susceptibility to biotrophic pathogens (Pan et al. 2016; Qiang et al. 2021). Belonging to the *A. thaliana MtN21* gene family, *RTP1* is similar to nodulin-related genes from *Medicago truncatula*. RTP1 contains 8 to 10 predicted transmembrane domains and localizes to endoplasmic reticulum (ER) membranes (Pan et al. 2016). Compared with wild-type (WT) *A. thaliana* plants, *rtp1* mutants are more resistant to the oomycete *P. parasitica*, the fungus *Golovinomyces cichoracearum*, and the bacterial pathogen *P. syringae* pv tomato (Pst) DC3000 (Pan et al. 2016). Furthermore, *rtp1* mutants show increased reactive oxygen species (ROS) production and accelerated defense gene expression (Pan et al. 2016), which are hallmarks of an immune response and indicate that RTP1 may be involved in negative regulation of plant defences.

Previously, it was reported that RTP1 acts on regulators of the unfolded protein response (UPR) (Qiang et al. 2021). Under stress conditions, such as pathogen infection, unfolded or misfolded proteins accumulate in the endoplasmic reticulum (ER). To resolve this situation and prevent cell death, plant cells activate signaling cascades collectively referred to as the UPR. The ER membrane–associated transcription factors bZIP28 and bZIP60 act as stress sensors in parallel signaling pathways to upregulate expression of UPR genes (Kwan Ko and Brandizzi 2024). Production of active bZIP60 is achieved by unconventional splicing of the *bZIP60* mRNA; increased splicing of *bZIP60* was observed in *rtp1* mutants, suggesting that RTP1 is a negative regulator of bZIP60 activation. In addition, RTP1 appears to interact with and stabilize bZIP28, possibly leading to increased retention in the ER and reduced translocation to the nucleus (Qiang et al. 2021).

In a recent study published in *Plant Physiology*, Wei et al. (Wei et al. 2025) probed the function of RTP1 in greater detail. Comparative analysis of the transcriptomes of *P. parasitica*-infected roots of *A. thaliana rtp1* mutants and WT plants revealed upregulation of several genes from the Cytochrome P450 family 71 (CYP71) in *rtp1* mutants compared with WT plants. Cytochrome P450 (CYP) proteins are a diverse family of heme-containing enzymes that catalyze a wide range of reactions (Xu et al. 2015). *CYP71* genes have been reported to have a function in pathogen resistance, with some known to be required for biosynthesis of the antimicrobial molecule camalexin. The authors focused on an uncharacterized member of the CYP71 family, CYP71B3, for further study. *A. thaliana cyp71b3* mutants were more susceptible than WT Col-0 plants to both the oomycete *P. parasitica* and the bacterial pathogen Pst DC3000. In contrast, CYP71B3 overexpressing lines were more resistant to both pathogens, suggesting that CYP71B3 is a positive regulator of plant resistance to biotrophic plant pathogens (Wei et al. 2025).

After demonstrating that, like RTP1, GFP-tagged CYP71B3 is localized in the ER, the authors used split-luciferase and coimmunoprecipitation assays to investigate whether RTP1 binds to CYP71B3. The results suggested that the two proteins interact. By coexpressing CYP71B3 in *N. benthamiana* with either RTP1 or an empty vector (EV) control, the authors observed reduced accumulation of CYP71B3 in the presence of RTP1 compared with EV and concluded that RTP1 destabilizes CYP71B3 (Wei et al. 2025).

The authors subsequently aimed to identify regions of CYP71B3 involved in interaction with RTP1. Comparison of CYP71B3 with other *A. thaliana* CYP71 family members identified I38 as a residue exclusively present in RTP1-interacting CYP71 proteins. Wei et al. hypothesized that I38 was therefore important for RTP1 binding. Introduction of an I38A mutation into CYP71B3 resulted in a loss of the luciferase complementation signal observed when WT CYP71B3 was coexpressed with RTP1. Furthermore, unlike WT CYP71B3, CYP71B3^I38A^ could not genetically complement *cyp71b3* mutants (Wei et al. 2025). However, it is yet to be determined whether CYP71B3^I38A^ lies at an RTP1 binding interface or whether this mutation affects the ER localization of the protein or alters its structure to render it nonfunctional.

Finally, the authors note that CYP71B3 expression is regulated by the transcription factor bZIP60 (Wei et al. 2025). The results presented here complement the previous study by Qiang et al. (2021) and collectively support a function for RTP1 as a regulator of UPR during biotic stress. As the precise enzymatic activity of CYP71B3 remains unknown, the metabolic consequences of upregulation of CYP71B3—and how this leads to enhanced resistance—remain to be explored.

Studies of susceptibility genes can give insights into how pathogens successfully cause disease. In addition, understanding at a molecular level how S genes condition host susceptibility opens new avenues for development of resistant crop varieties. While some S genes have been successfully deployed in the field, in other cases, knocking out S genes leads to pleiotropic phenotypes that prevent their use in an agricultural context (Garcia-Ruiz et al. 2021). Insights into the precise function of S gene products could enable targeted modification to alter their activity, expression, or interactions with other proteins (Garcia-Ruiz et al. 2021).

In summary, the work presented here extends our understanding of the role of RTP1 in modulating host plant susceptibility to biotrophic pathogens and implicates the previously uncharacterized CYP71B3 protein in regulating plant biotic stress responses (Figure). Functional characterisation of S genes provides insight into pathogen infection requirements and opportunities to develop novel genetic resistance to crop pathogens.

**Figure.**
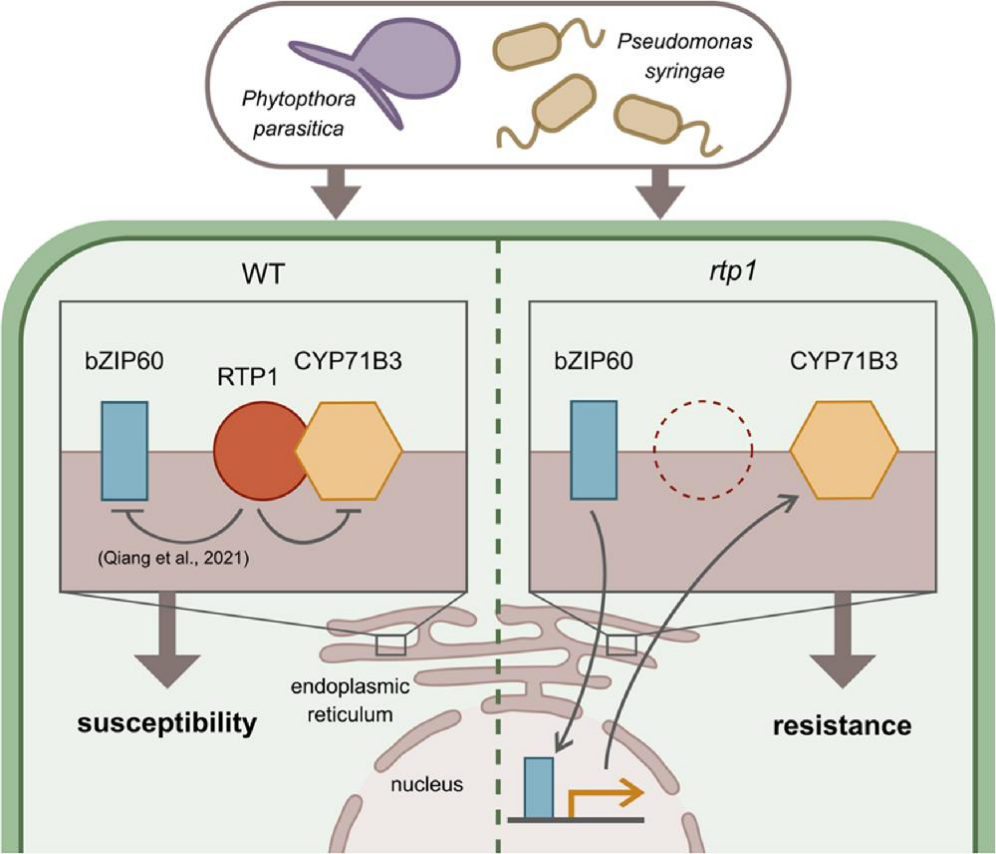
Schematic model (adapted from Wei et al. 2025) summarizing the contributions of RTP1, CYP71B3, and bZIP60 to susceptibility or resistance in *A. thaliana* WT (left) or *rtp1* (right) plants. Left: In WT *A. thaliana* plants RTP1 interacts with and destabilizes CYP71B3. RTP1 has previously been reported to negatively regulate bZIP60 by modulating splicing required for production of active bZIP60 (Qiang et al. 2021). The activity of RTP1 leads to host plant susceptibility to the biotrophic pathogens *P. parasitica* and *P. syringae*. Right: In *rtp1* mutants, active bZIP60 upregulates CYP71B3 expression and CYP71B3 is no longer destabilized by RTP1, leading to increased resistance to *P. parasitica* and *P. syringae*.

## Data Availability

No new data were generated or analysed for this article.
